# Free-living competitive racewalkers and runners with energy availability estimates of <35 kcal·kg fat-free mass^−1^·day^−1^ exhibit peak serum progesterone concentrations indicative of ovulatory disturbances: a pilot study

**DOI:** 10.3389/fspor.2023.1279534

**Published:** 2023-11-17

**Authors:** M. Carolina Castellanos-Mendoza, Stuart D. R. Galloway, Oliver C. Witard

**Affiliations:** ^1^ Physiology, Exercise and Nutrition Research Group, Faculty of Health Sciences and Sport, University of Stirling, Stirling, United Kingdom; ^2^ Centre for Human and Applied Physiological Sciences, Faculty of Life Sciences and Medicine, King’s College London, London, United Kingdom

**Keywords:** anovulation, luteal phase deficiency, short luteal phase, female athletes, endurance sports, menstrual cycle, exercise, energy availability

## Abstract

**Introduction:**

The release of luteinising hormone (LH) before ovulation is disrupted during a state of low energy availability (EA). However, it remains unknown whether a threshold EA exists in athletic populations to trigger ovulatory disturbances (anovulation and luteal phase deficiency) as indicated by peak/mid-luteal serum progesterone concentration (Pk-PRG) during the menstrual cycle.

**Methods:**

We assessed EA and Pk-PRG in 15 menstrual cycles to investigate the relationship between EA and Pk-PRG in free-living, competitive (trained-elite) Guatemalan racewalkers (*n *= 8) and runners (*n *= 7) [aged: 20 (14–41) years; post-menarche: 5 (2–26) years; height: 1.53 ± 0.09 m; mass: 49 ± 6 kg (41 ± 5 kg fat-free mass “FFM”)]. EA was estimated over 7 consecutive days within the follicular phase using food, training, and physical activity diaries. A fasted blood sample was collected during the Pk-PRG period, 6–8 days after the LH peak, but before the final 2 days of each cycle. Serum progesterone concentration was quantified using electrochemiluminescence immunoassay.

**Results:**

Participants that reported an EA of <35 kcal·kg FFM^−1^·day^−1^ (*n *= 7) exhibited ovulatory disturbances (Pk-PRG ≤9.40 ng·mL^−1^). Athletes with EA ≥36 kcal·kg FFM^−1^·day^−1^ (*n *= 8) recorded “normal”/“potentially fertile” cycles (Pk-PRG >9.40 ng·mL^−1^), except for a single racewalker with the lowest reported protein intake (1.1 g·kg body mass^−1^·day^−1^). EA was positively associated with Pk-PRG [*r*(9) = 0.79, 95% confidence interval (CI): 0.37–0.94; *p *= 0.003; 1 − *β* = 0.99] after excluding participants (*n *= 4) that likely under-reported/reduced their dietary intake.

**Conclusions:**

The result from the linear regression analysis suggests that an EA ≥ 36 kcal·kg FFM^−1^·day^−1^ is required to achieve “normal ovulation.” The threshold EA associated with ovulatory disturbances in athletes and non-invasive means of monitoring the ovulatory status warrant further research.

## 1. Introduction

Energy availability (EA) is a concept in sports nutrition developed by Loucks et al. (1) to represent dietary energy intake (EI) available to support all physiological processes and human health. Accordingly, EA is calculated by subtracting the total energy cost of exercise in surplus of non-exercise waking activity [exercise energy expenditure (EEE)] from EI and then normalising to individual fat-free mass (FFM). Hence, the unit of expression for EA is kcal·kg FFM^−1^·day^−1^. Healthy females typically achieve energy balance at ≈45 kcal·kg FFM^−1^·day^−1^ (2). However, restricting EA to 30 kcal·kg FFM^−1^·day^−1^ results in a decline in biomarkers of bone formation (3) and hormonal changes, specifically a decrease in the concentration of insulin, triiodothyronine, and leptin and an increase in the concentration of cortisol (4).

The pulsatile release of luteinising hormone (LH) is disrupted at a threshold EA of <30 kcal·kg FFM^−1^·day^−1^ (4). Moreover, the concentration of follicle-stimulating hormone is increased as EA declines to 10 kcal·kg FFM^−1^·day^−1^, although this trend is reported only if EA restriction is caused by EEE (1). Furthermore, bone resorption is increased at an EA of 10 kcal·kg FFM^−1^·day^−1^ (3). These physiological changes were documented in young women after 4–5 days of EA restriction under controlled laboratory conditions (1, 3, 4). Since estimates of self-reported EA are prone to error, research conducted on free-living athletes has failed to determine thresholds or associations between EA and disruptions to metabolic hormones (5) or ovulatory disturbances (6), i.e., anovulation and luteal (post-ovulatory) phase deficiency. Hence, carefully designed studies are warranted to fill this gap in knowledge.

The surge in LH concentration stimulates ovulation (7), and the ovarian follicle responsible for releasing the ovum develops into a transient gland that mainly produces progesterone (8). “Ovulation” is assumed with a serum progesterone concentration of ≥3.0 ng·mL^−1^ [≥9.54 nmol·L^−1^ (9)] or a peak concentration of >6.0 ng·mL^−1^ (10). Nevertheless, ovulatory cycles may exhibit “luteal phase deficiency or defect,” defined as a serum progesterone concentration of <5.0 ng·mL^−1^ assessed at any timepoint during the luteal phase (11). The luteal phase is typically ∼14 days in duration, regardless of the length of the menstrual cycle (7). Luteal phase deficiency exhibited as “late ovulation” or “short luteal phase” (<10 days as of the second day after the LH peak) is associated with a peak serum progesterone concentration of <10.0 ng·mL^−1^ (12). In contrast, a single mid-luteal serum progesterone concentration of >9.4 ng·mL^−1^ indicates a “potentially fertile” cycle (13).

Ovulatory menstrual cycles have lower bone resorption rates during their luteal phase compared with anovulatory cycles (10), but optimum peak progesterone concentrations for bone health remain to be fully elucidated. When the oestradiol status is maintained, luteal phase defects cause no apparent change in bone health after 3 months (14). However, during a 1-year follow-up, ≥2 cycles with a “short luteal phase” [<10 days by basal body temperature (BBT) quantitative interpretation] are associated with a decline in bone mineral density, with women exhibiting anovulation more prone to greater spinal bone loss (15). This association between frequent ovulatory disturbances and negative changes in bone mass has been confirmed in several prospective studies (16). Moreover, bone health, menstrual function, and EA constitute a triad (17) within a host of health issues characterised by the syndrome of Relative Energy Deficiency in Sport or “RED-S” (18), as observed in athletes that chronically fail to meet energy demands. Athletes in a state of low energy availability (LEA), defined as <30 kcal·kg FFM^−1^·day^−1^, that experience menstrual disturbances, i.e., oligomenorrhoea or amenorrhoea, often exhibit a lower resting metabolic rate (RMR) than eumenorrheic athletes who report adequate EA, i.e., ≥45 kcal·kg FFM^−1^·day^−1^ (19). Interestingly, the frequency of injury is greater in athletes with menstrual disturbances (20), while female endurance athletes with symptoms of LEA are at higher risk of developing bone stress injury due to exhibiting poor bone health (21). Accordingly, with regard to long-term health and performance in female athletes, energy restriction should not trigger anovulation or ≥2 “short luteal phases” per year (15). Nonetheless, whether a threshold exists for the association of EA with ovulatory disturbances remains unknown.

By design, female runners (22–24) and racewalkers (22) are frequently in a state of LEA and exhibit ovulatory disturbances (14, 15, 25), especially when failing to increase EI with training overload (26). However, to our knowledge, no study has investigated the association between EA and peak progesterone concentration or identified the threshold of EA that compromises fertility [mid-luteal serum progesterone ≤9.4 ng·mL^−1^ (13)]. Therefore, the primary aim of this study is to estimate EA during free-living conditions using a field-based methodology and explore the relationship between EA and subsequent peak progesterone concentration in competitive racewalkers and runners that were not using hormonal contraception.

## 2. Materials and methods

This study received ethical approval for invasive research in human participants from the NHS, Invasive or Clinical Research (NICR) Committee at the University of Stirling (1 June 2017, NICR 16/17—Paper No. 58) and local endorsements from three sports institutions in Guatemala (refer to Ethics statement).

### 2.1. Eligibility and recruitment

The Low Energy Availability in Females Questionnaire (LEAF-Q) was used to determine study eligibility: score <8 points, “not at risk of LEA” (27). The criteria included participants that self-reported being non-smokers, not pregnant or lactating, not taking medications associated with any chronic disease, ≥2 years post-menarche, without signs or symptoms of perimenopause, not using hormonal contraception during the preceding 6 months, and “naturally menstruating” (11) in three previous menstrual cycles. A total of 34 eligible athletes were informed regarding this study through the cooperation of coaches and staff members from the National Athletics Federation. In total, 28 Guatemalan racewalkers and runners voluntarily agreed to participate in this research, and provided informed consent prior to their involvement. However, only 26 athletes started the study.

### 2.2. Study design and data collection

**Figure 1:**
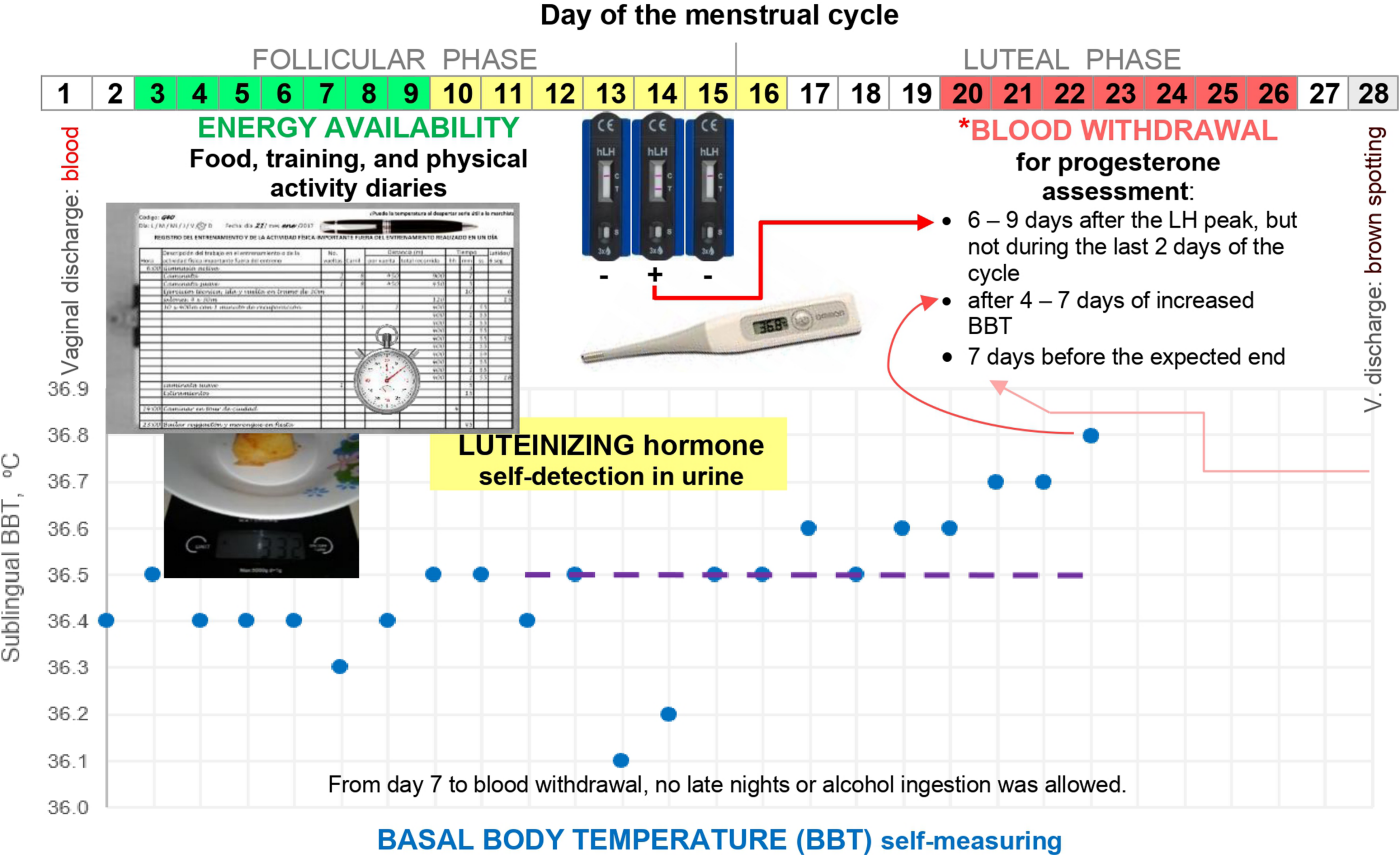
Overview of the study protocol during a 28-day menstrual cycle. *Points of reference are stated in order of priority. The shift from low to high BBT, from day 15 to 16, is doubtful at the beginning because BBT was back to the previous lower BBT level on day 17. BBT was measured immediately upon awakening after a night-time sleep, while still in bed. BBT log included remarks about vaginal discharge to define cycle length and factors that could alter BBT such as alcohol ingestion, signs and symptoms of infections, duration and quality of sleep, use of medications, and unusual environment or stress (28).

Figure 1 summarises the study protocol and illustrates the timing of assessments during each menstrual cycle. Researchers explained all data collection procedures and monitored athletes in person and via chat apps or phone calls. Prospective observational data were collected under free-living conditions.

### 2.3. Basal body temperature

Athletes conducted daily measurements of sublingual BBT using a digital thermometer (Omron MC−343F) with an accuracy of 0.1°C. The BBT chart was tracked throughout the menstrual cycle (Figure 1). A female is assumed to have ovulated after observing 3 consecutive days of elevated BBT measurements (28) that are higher than those of the previous 6 days (Sensiplan® rules for quantitative interpretation of BBT requires further verification of algorithms to confirm ovulation). This shift from lower to higher BBT provided a benchmark to schedule progesterone assessment if the day of LH peak was missed, i.e., day 1 of elevated BBT reflected day 1 of the luteal or “high-BBT” phase.

### 2.4. Luteinising hormone detection or “ovulation testing”

Participants used the hLH Cassette 002L040 (UltiMed™, Germany) to self-detect LH peak concentrations in urine following manufacturer instructions. This rapid-chromatographic-immunoassay test detects LH only and not LH metabolites. First-morning urine was not assayed because it could miss the LH peak (29). Moreover, given that most athletes are under the time pressure of training or school in the morning, volunteers conducted this test during the expected peak of LH (Figure 1) once a day at 8 p.m. or 10 a.m. if forgotten the night before. The first author notified the participants individually when to start testing, e.g., day 11 if expecting a 28-day cycle or earlier/later for shorter/longer cycles. Qualitative detection of LH [≥30 mIU·ml^−1^] continued until the day after a positive result.

Given that surges in LH concentration vary in amplitude, duration, and peak configuration (single, double, multiple, or plateau), the day of ovulation as determined by ultrasound may occur at the onset or end, during, or after the LH peak (30). However, ovulation occurs on the day (15%), the day after (76%), or ≥2 days after the first detection (9%) and not before urinary detection of LH >30 IU·L^−1^ (31). During cycles with long LH surges, higher BBT measurements begin the day after ovulation [Supplemental Figure 2B in Direito et al. (30)]. Therefore, we define the “day of LH peak” as the last consecutive day with a positive detection of LH and the presumed ovulation day as the final day of the follicular phase. We adhere to the definition of Schliep et al. (12) for the “presumed ovulation day” as the day of LH peak plus 1 day. LH was positive for two consecutive days in three menstrual cycles. In two of these cycles, the luteal phase length was the same as the high-BBT phase length.

### 2.5. Progesterone quantification

The participants involved in the study resided in four different cities. Two accredited laboratories (*Centro Médico* and *TecniScan*) determined serum progesterone concentrations using electrochemiluminescence immunoassay with an automated Cobas e601 analyser (Roche Diagnostics). Identical results were obtained for extremely low progesterone concentration, although differences of 1.01–1.10 ng·mL^−1^ were observed between laboratories for duplicate analysis of samples with intermediate and high concentrations. The average peak progesterone concentration of duplicates was used in the data analysis. To minimise participant burden, we planned blood withdrawal once within the expected progesterone peak period of each cycle. The athletes were encouraged to be euhydrated for blood sampling that was scheduled at 7 a.m. in an overnight fasted state. Blood vacutainers were centrifuged to separate the serum and were refrigerated until further analysis within 48 h.

“Peak progesterone” refers to a concentration quantified 6–9 days after the day of LH peak, but before the final 2 days of the cycle (10). In line with this definition, we documented progesterone concentration 6–8 days after the day of LH peak for six participants. Despite missing the LH peak (*n* = 6), progesterone concentration was quantified during days 5–8 of the high-BBT phase, within the mid-luteal period defined by BBT interpretation (13) and without statistical differences in the timing of assessment between groups of cycles by ovulatory status (Table 1). For cases with no LH surge during expected peak and progesterone concentrations indicative of anovulation, we verified that quantification occurred within the period before the end of the cycle with expected high concentrations (13). The peak, mid-luteal, and progesterone concentrations quantified during the expected peak are all indicative of the ovulatory status of a menstrual cycle. Therefore, we classified ovulatory status with progesterone concentrations as follows: “anovulatory” if ≤6.00 ng·mL^−1^ (10), “luteal phase defect” if 6.01–9.40 ng·mL^−1^, and “potentially fertile” or “normal ovulatory” if >9.40 ng·mL^−1^ (13). Three cycles had peak (*n *= 2) or mid-luteal (*n* = 1) progesterone concentrations that approached the critical cut-off point between “ovulatory disturbed” and “potentially fertile” (9.00–10.00 ng·mL^−1^). We highlight that the Quantitative Basal Temperature (QBT) method (32) and Sensiplan® rules were both consistent with the progesterone concentrations in classifying these cycles as “luteal phase defect” (*n* = 2) or “normal ovulatory” (*n* = 1).

**Table 1:** Details and timing of assessments during menstrual cycles.

	*n*	Timing of assessment		
The 7-day period of energy availability estimation ended before expected ovulation, specifically on:				
	3	Day of the anovulatory cycles		10 ± 2, 9–12
	6	Days before the “day of LH peak”		6 ± 3, 2–9
	6	Days before the “last day of the follicular phase” by BBT		9 ± 3, 6–14
LH				
Not detected	2	Progesterone during the expected peak period by cycle length indicative of anovulation		
False positive^a^	1			
Detection, LH+	6	On 1 day only (*n* = 3), for 2 consecutive days (*n* = 3)		
Missed detection^b^	6	Progesterone during the peak period by BBT indicative of ovulation		
Day of LH peak	6	Day of the cycle		16 ± 3, 12–21
Serum progesterone quantification^c^	6	By *Centro Médico* (CM)		
	8	By *TecniScan* (TS)		
	1	By both laboratories		
	15	Day of the cycle		24 ± 3, 19–32
	6	Days after the “day of LH peak”		8 (6–8)
	6	Day of the luteal (post-ovulatory) phase by LH peak		7 (5–7)
	6	Day of the high-BBT phase^d^ if LH detection was missed		6 ± 1, 5–8
	15	Days before the last day of the cycle		5 (4–10)
Timing of serum progesterone (PRG) quantification^e^				
	Ovulatory status			
	Anovulatory	Luteal phase defect	Potentially fertile	High PRG
Progesterone concentration cut-offs, ng·mL^−1^	<6.00	6.00–9.40	9.41–16.00	>16.00
*N*	3	5	4	3
Day of the cycle	23 ± 3	24 ± 2	24 ± 1	26 ± 7
	21–27	21–27	23–25	19–32
Days after the “day of LH peak”	7	8 (6–8)	8	6
	(false LH+)	*n* = 4	*n* = 1	*n* = 1
Day of the luteal phase, defined by LH peak		7 (5–7)	7	5
		*n* = 4	*n* = 1	*n* = 1
Day of the high-BBT phase (*n* = missed LH detection)		5^f^	6 ± 1, 5–8^f^	6^f^
		*n* = 1	*n* = 3	*n* = 2
Days before the last day of the cycle	5 (5–8)^f^	6 (4–10)	7 (4–9)	5 (5–9)

LH, Luteinizing hormone. Day of LH peak: last consecutive day with positive urinary detection of LH (LH+). BBT, basal body temperature. Last day of the follicular phase: day of LH peak + 1 day (12) or the last day of the “low-BBT phase” by BBT quantitative interpretation.

^a^ Progesterone of 2.89 ng·mL^−1^ was quantified 5 days before the last day of the cycle on day 7 after LH+, thus false LH+ for ovulation.

^b^ A participant forgot to test on the day that could have been LH+, the others (*n* = 5) had shorter or longer cycles than expected.

^c^ CM quantified 0.62, 11.13, and 22.82, while TS quantified, respectively, 0.62, 10.03, and 23.83 ng·mL^−1^.

^d^ High-BBT phase length was defined by Sensiplan® rules (*n* = 5) and the Quantitative Basal Temperature “QBT” method (*n* = 1).

^e^ There was no significant difference in progesterone quantification timing between groups of cycles classified by their ovulatory status.

^f^ Within the period of expected high progesterone concentrations and within the mid-luteal period for the ovulatory (13).

### 2.6. Energy availability

Prior to expected ovulation and within days 3–12 of the menstrual cycle, the food diaries, training diaries, and physical activity questionnaires were completed over 7 consecutive days to estimate EA based on the 7-day average of both EI and EEE (refer to Supplementary Table S1 for data collection details). A 7-day period represents the repetitive lifestyle pattern and a complete training micro-cycle, including all types of workouts and 1 day of rest over the weekend whereas a longer period was considered too onerous (33). The estimation period for EA occurred prior to the day of the LH peak or the end of the follicular phase as shown by the quantitative interpretation of BBT (Table 1). If an athlete reported any difference between the first and second weeks of the studied cycle in terms of (i) dietary pattern due to travelling or festivities likely changing EI, (ii) amount of regular food consumed, or (iii) training volume (intensified or tapered), the estimated EA was deemed non-representative of the follicular phase, and the cycle was discarded from the analysis.

#### 2.6.1. Dietary assessment

The participants recorded a weighed and photographed food diary that included the data on family meal recipes with the consumed proportion. All athletes weighed their food, except for two runners who reported portion sizes using measuring cups and spoons. If unable to weigh food (unplanned eating), the photograph and description of a meal or snack were used to estimate the portion size and weight. To ensure valid and accurate data, an accredited Sports Dietitian (first author) interviewed the athletes daily and within 7 days following the dietary register week and checked all food items for their code and weight to confirm the agreement with the portion reported or photographed prior to conducting dietary analysis with NutrINCAP® (version 2.1) software. NutrINCAP® uses the Food Composition Tables of Central America, with the possibility to incorporate data for additional products. Data were verified by double-checking the records of days with low or high energy or nutrient intakes. The Diet Quality Index International (DQI-I) score (34) was estimated.

#### 2.6.2. Exercise “training and physical activity” energy expenditure

The first author and assistant researchers documented the training in printed form by observation or, otherwise, it was self-reported by the athlete. The participants also detailed their physical activity outside of training to the nearest minute. The first author assigned metabolic equivalent of tasks (METs) value for each physical activity after interviewing the athlete to verify the accuracy of self-reported information or observations made by assistant researchers. If METs data were unavailable for adolescents, i.e., running >12.9 km·h^−1^, the adult value was used (Table 2). After data tabulation, EEE was estimated using Excel® 365. EEE was calculated as the total energy cost of training and physical activity minus the energy cost of being awake but not exercising over the same period (see non-exercise energy cost below).

**Table 2:** Equations to estimate energy availability.

Abbreviations	Equation or definition	Units
FFM: fat-free mass	FFM=bodymass−bodyfatmass ^a^	kg
RMR: resting metabolic rate		
RMR, female adult aged ≥18 years:	Harris and Benedict (35)	$\frac{\mathrm{kcal}}{\mathrm{day}}$
RMR, female adolescent aged 14–17.9 years:	^b^Schofield (36) − 5%	
RMR per minute	$\frac{\mathrm{RMR}}{\min}=\;\mathrm{RMR}\;\cdot \;\frac{1\;\mathrm{day}}{1440\;\min}$	$\frac{\mathrm{kcal}}{\min}$
METs: metabolic equivalent of tasks	Databases. Adult: Ainsworth et al. (37). Adolescent: Butte et al. (38).	
TPA: training + physical activity	activity ≥4 METs + 3.5–3.9 METs if completed for ≥10 min·day^−1^	
^c^TECsA: total energy cost of a specific activity (sA)	$\mathrm{TECsA}=(\mathrm{MET}\;\mathrm{sA})\frac{\mathrm{RMR}}{\min}(\min\mathrm{sA})$	kcal
TECE: total energy cost of exercise “TPA”	TECE=ΣTECsA	kcal
^d^ECBANE: energy cost of being awake but not exercising, during the time engaged in TPA	$\mathrm{ECBANE}=1.3\;\;\cdot \;\;\frac{\mathrm{RMR}}{\min}\;\;\cdot \;\;\frac{\min\mathrm{TPA}}{\mathrm{day}}$	$\frac{\mathrm{kcal}}{\mathrm{day}}$
EEE: exercise “TPA” energy expenditure	EEE=TECE−ECBANE	$\frac{\mathrm{kcal}}{\mathrm{day}}$
EA: energy availability EI: dietary energy intake	$\mathrm{EA}=\frac{\mathrm{EI}-\mathrm{EEE}}{\mathrm{FFM}}$	$\frac{\mathrm{kcal}}{\mathrm{kg}\;\mathrm{FFM}\cdot \mathrm{day}}$

^a^ Body fat percentage, to estimate body fat mass, as per the equation in Yuhasz (39) that requires 6 skinfolds.

^b^ Predicts RMR from body mass.

^c^ As per Butte et al. (38).

^d^ Factor 1.3 for adults, but 1.33 for adolescents.

##### 2.6.2.1. Resting metabolic rate

Due to a lack of validated equations for our specific athletic population, we chose the Harris and Benedict (35) equation to estimate RMR in adult participants, as it predicts RMR in female athletes (40) including sports that predispose a low body mass type (41). The Schofield (36) equation that predicts RMR from body mass was used in our adolescent participants with a 5% correction, as suggested by the Institute of Nutrition of Central America and Panama “INCAP” (42).

##### 2.6.2.2. Non-exercise energy cost

The non-exercise energy cost was defined as 1.3 × RMR per minute, multiplied by the time engaged in training and physical activity (Table 2). This conversion factor is based on the estimated energy cost of a 10-h rest plus a 14-h very light activity in adults (43). This value was substituted in adolescents for 1.33, which was estimated with data from Torún et al. (42) using the same rest-activity ratio and accounting for growth energy estimates in females aged 14–17.9 years.

##### 2.6.2.3. Total energy cost of exercise “training and physical activity”

The total energy cost of exercise was estimated from the data analysis of training diaries, excluding passive stretching, and physical activity questionnaires. Physical activity was defined as efforts ≥3.5 METs. The data regarding non-training activities ≥4 METs (i.e., dancing, physical-household chores such as wood piling, biking, or walking for transportation, carrying a backpack or child) were included in the estimation of EEE (44). Moderate household chores equivalent to 3.5–3.9 METs (i.e., floor or bathroom cleaning) were computed if completed for ≥10 min per day. METs were used as a multiple of individual RMR (Table 2). Regular walking, running, and corrected running METs were used to estimate the total energy cost of racewalking according to speed (Table 3).

**Table 3:** Metabolic equivalents of tasks (METs) used to estimate the total cost of exercise.

Racewalking		
speed, km·h^−1^	METs	Assumed due to:
<8.0	Regular walking	Hagber and Coyle (45)
8.0–9.7	Running	
> 9.7	Running + 1.6	“1.6 METs”: oxygen consumption data of highly-trained male and female racewalkers in Mora-Rodriguez et al. (46) comparing running and racewalking at 10.9 km·h^−1^
Typical training activities^a^		
Running or racewalking	0.2^b^	Flat surface with ∼1 kg around each wrist
	0.3^b^	Uphill
	0.4^b^	Uphill (very steep)
	0.5^b^	Flat surface wearing a vest of at least 5 kg
	4.0	Continuously active gymnastics or callisthenics Isometric exercises, e.g., maintaining tough yoga positions
	5.0	Technique drills or multiple jump exercises Resistance exercises, moderate effort
	6.0	Tough resistance exercises with own body mass (e.g., pull-ups, push-ups), high intensity
	8.0	Gym circuit, severe effort, and minimal rest

^a^ We used heart rate values upon task completion to assign METs for specific activities not included in databases, e.g., heart rates immediately after “technique drills” and “resistance exercises–moderate effort” were similar.

^b^ Amounts added to the specific METs: determined from the adult database (37); e.g., the difference between walking or running at a specific speed on “inclined” and “flat” surfaces.

#### 2.6.3. Body composition

FFM was estimated during the previous or studied menstrual cycle using a two-compartment body composition model with anthropometry [equation proposed by Yuhasz (39)]. All measurements were conducted before the first training session using the International Society for the Advancement of Kinanthropometry (ISAK) methodology by the same qualified anthropometry practitioner (Supplementary Table S1). The assessment was not undertaken before or during menstruation (vaginal discharge of the inner lining of the uterus) when self-reported scores for fluid retention or bloating (puffiness + oedema + nocturia) are typically highest (47). The technical error of measurement of skinfolds used to estimate body fat percentage was ≤3.6%.

### 2.7. Ovulatory status prior to study

The participants were not required to be “eumenorrheic” (11). Two athletes recorded their BBT during the menstrual cycle prior to participation in the study. Several participants voluntarily repeated the study during the following cycle as data generated in the first attempt were discarded from the analysis as explained in Supplementary Table S2. Therefore, we documented the ovulatory status of the menstrual cycle prior to this study in six participants as per progesterone (*n* = 3), BBT (*n* = 2), and LH (*n* = 1).

### 2.8. Statistical analysis

EI relative to the measured RMR (EI:mRMR) <1.35 has been recognised as incompatible with long-term survival (48) and is typically used as an indicator of presumed EI underreporting (44). Although RMR was not measured, EI was the average of only 7 days, and ovulatory disturbances were expected (14, 15, 25), particularly if associated with reports of low EI. Hence, we chose to present data for all volunteers but conduct correlation analysis excluding four participants with EI relative to estimated RMR (EI:eRMR) <1.35. The relationship between EA and peak/mid-luteal progesterone was analysed using Pearson's correlation coefficient, *r*(*n*−2), with estimated 95% confidence interval (CI). We used a regression analysis to determine the EA required by our participants to achieve fertile progesterone concentrations.

Descriptive statistics are presented as mean ± SD (including range if the data set is skewed and if minimum and maximum values are critical) or median (range) if not normally distributed as per Shapiro–Wilk test (IBM©-SPSS®). We compared three “anovulatory” cycles with those exhibiting the three highest progesterone concentrations, introducing an additional ovulatory status group: “high progesterone” (>16.0 ng·mL^−1^). One-way analysis of variance (ANOVA) with Tukey's HSD *post-hoc* test was conducted to investigate the differences in EA between menstrual cycles with “anovulatory,” “luteal phase defect,” “potentially fertile,” and “high” peak/mid-luteal progesterone and also to explore the differences in variables among groups of participants classified by ovulatory status. The data of a variable were presented and analysed non-parametrically if it was not normally distributed in one or more of these groups. Kruskal–Wallis with Dunn's *post-hoc* test was used as the non-parametric alternative. Significance level for all tests was set at an *α* = 0.05. The *post-hoc* power (1 − *β*) was estimated using G*Power 3.1.9.4 (49).

## 3. Results

Two of the 26 volunteers who started this study dropped out. We failed to quantify the progesterone concentration within the peak period for five athletes. The ovulatory status of 19 participants was recorded based on their progesterone concentration, but four cases were excluded. The lost cases and final exclusions are described in Supplementary Table S2.

### 3.1. Participants

The final data set consisted of competitive racewalkers (*n* = 8) and runners (*n* = 7), 5 (2–26) years post-menarche, with training and performance classification (50) from trained (tier 2) to elite/international level (tier 4). The descriptive characteristics of the participants are shown in Table 4. A total of 15 menstrual cycles were examined, with each participant contributing one cycle for analysis (Table 5).

**Table 4:** Characteristics, body composition, training, and physical activity of participants.

	Ovulatory status				
	Anovulatory	Luteal phase defect	Potentially fertile	High PRG	Total
Serum progesterone (PRG) cut-offs, ng·mL^−1^	<6.00	6.00–9.40	9.41–16.00	>16.00	* *
*N*	3	5	4	3	15
Characteristics					
Rw: racewalkers, *n* R: runners, *n*	Rw = 2	Rw = 2	Rw = 3	Rw = 1	Rw = 8
	R = 1	R = 3	R = 1	R = 2	R = 7
Training and performance calibre,^a^ *n* per tier	2: *n* = 1	2: *n* = 4	2: *n* = 1	2: *n* = 1	2: *n* = 7
	3: *n* = 2	3: *n* = 1	3: *n* = 3	3: *n* = 1	3: *n* = 7
				4: *n* = 1	4: *n* = 1
Chronological age, years	18	18	24	20	20
	(16–20)	(14–21)	(17–41)	(19–22)	(14.8–41.1)
Gynaecological age, years post-menarche	3	4	12	6	5
	(1.7–5.0)	(2–8)	(4–26)	(4–9)	(1.7–26.1)
LEAF-Q score,^b^ points	6	4	5	6	5
	(3–7)	(0–5)	(1–9)	(2–8)	(0–9)
Anthropometry and body composition					
Body mass, kg	48.2 ± 0.6	49.2 ± 5.3	50.3 ± 8.1	46.3 ± 7.6	49 ± 6 40–61
Height, m	1.53 ± 0.01	1.49 ± 0.04	1.54 ± 0.14	1.55 ± 0.15	1.53 ± 0.09 1.39–1.74
Body mass index, kg·m^−2^	20.5	20.6	21.0	19.1	20.5
	(20.5–21.1)	(19.4–25.9)	(20.2–21.9)	(18.0–20.4)	(18.0–25.9)
Sum of 8 skinfolds, mm		* ’	’	*	
	106	110	82	83	96
	(90–114)	(96–162)	(80–101)	(71–87)	(71–162)
Body fat, % as per Yuhasz (39)		* ’	’	*	
	15	16	13	14	15
	(15–17)	(15–22)	(13–16)	(12–14)	(12–22)
Training and physical activity “TPA” ≥ 3.5 metabolic equivalent of tasks (METs)					
Volume, km per week	62	23	62	44	44
	(40–103)	(12–90)	(18–92)	(26–60)	(12–103)
Volume, h per week	6	5	9	5	6
	(5–23)	(3–12)	(5–14)	(4–8)	(3–23)
EEE: exercise “TPA” energy expenditure, kcal per day	493	195	443	388	385
	(318–690)	(150–572)	(204–696)	(189–394)	(150–696)

Significant differences between groups: * and ‘ *p *< 0.05.

^a^ As per McKay et al. (50); tier 2: local level representation; tier 3: competitive athletes at both the National (Guatemala) and Central American levels.

^b^ LEAF-Q: Low Energy Availability (LEA) in Females Questionnaire, score ≥8 points = risk for LEA (27). Two athletes with 8 and 9 points were accepted as scores reflected respectively 3-week absence of training (posterior tibial syndrome) and postpartum amenorrhoea.

**Table 5:** Diet and menstrual cycles of participants.

	Ovulatory status				
	Anovulatory	Luteal phase defect	Potentially fertile	High PRG	Total
Serum progesterone (PRG) cut-offs, ng·mL^−1^	<6.00	6.00–9.40	9.41–16.00	>16.00	* *
*N*	3	5	4	3	15
Reported dietary intake, 7-day average					
Energy (EI), kcal		*	*		
	1,756 ± 265	1,583 ± 202	2,096 ± 282	1,982 ± 174	1,834 ± 302 1,329–2,349
Energy, kcal per kg body mass		* ’	*	’	
	36 ± 6	32 ± 3	42 ± 4	43 ± 3	38 ± 6; 28–46
EI:_e_RMR	** **	**	**	** **	
	1.36 ± 0.21	1.23 ± 0.14	1.62 ± 0.16	1.54 ± 0.02	1.42 ± 0.22; 1.06–1.81
EI:_e_RMR < 1.35, *n*	1	3	0	0	4
Protein, g per kg body mass	** **	* **	**	*	
	1.1 (0.9–1.3)	1.0 (0.8–1.1)	1.4 (1.4–1.7)	1.3 (1.2–1.7)	1.2 (0.8–1.7)
Carbohydrate, g per kg body mass	5.4 ± 1.5	4.9 ± 1.0	6.4 ± 1.1	6.6 ± 1.1	5.7 ± 1.3; 4.0–7.7
Carbohydrate, g per kg fat-free mass	5.6 (5.3–8.5)	5.4 (4.7–7.4)	7.3 (5.9–9.1)	7.8 (6.3–8.7)	6.7 (4.7–9.1)
Energy from refined sugars,^a^ % kcal	* **	’	** ’	*	
	14.4 ± 4.2	10.7 ± 2.8	4.6 ± 1.3	6.8 ± 3.2	9.0 ± 4.5; 3.1–19.2
Fibre, grams	*	**	* **	** **	
	12 (12–14)	11 (10–15)	25 (17–28)	16 (9–17)	14 (9–28)
Diet quality index international “DQI-I,” points	64 ± 5	62 ± 8	73 ± 7	69 ± 13	67 ± 9; 50–82
Menstrual cycle					
Length, days	29 ± 3	31 ± 2	31 ± 3	32 ± 5	31 ± 3
	27–32	27–33	28–34	28–37	27–37
Luteal phase length by LH detection in urine (≥30 mIU·mL^−1^), days		12 ± 2			13 ± 2
		9–13	15	14	9–15
		*n* = 4^b^	*n* = 1	*n* = 1	*n* = 6
High-BBT phase length by Sensiplan® rules, days (*n* = missed LH detection)			11 ± 2		12 ± 2
		15	10–13	11	10–15
		*n* = 1^b^	*n* = 3	*n* = 2	*n* = 6
Serum progesterone, ng·mL^−1^	* **	* “	**	** “	10.34 ± 5.93
	2.96 ± 2.58	8.64 ± 0.69	10.90 ± 1.38	19.83 ± 3.34	
	0.41–5.57	7.63–9.36	9.58–12.84	16.09–22.51	

eRMR, estimated resting metabolic rate.

Significant differences between groups: * and ‘ *p *< 0.05 or ** and “ *p *< 0.01.

^a^ Reported dietary energy from added table sugar and refined sugars within commercial food, drinks, and candy.

^b^ High BBT phase length was indicated by the Quantitative Basal Temperature “QBT” method; PRG was quantified 10 days before the last day; Sensiplan® rules and QBT method indicated both a “luteal phase deficiency.”

The ovulatory status of nine athletes prior to this study was unknown. In six participants, the menstrual cycle prior to this study exhibited normal ovulation based on BBT interpretation (*n* = 1) and peak progesterone concentration (*n* = 1) and ovulatory disturbances based on mid-luteal progesterone (*n* = 2), BBT (*n* = 1), and LH (*n* = 1) measurements. The ovulatory status remained constant during the study in four athletes, whereas a marginal change was reported in two participants, i.e., luteal phase defect into either anovulatory or potentially fertile.

### 3.2. Ovulatory status

Eight menstrual cycles that were considered normal in length displayed “ovulatory disturbances”: “anovulation” (*n* = 3), “short luteal phase” (12) (*n* = 1), and LH peak at expected timing during the cycle but with progesterone concentration indicative of “luteal phase deficiency” (*n* = 4). The cycles with progesterone indicative of anovulation were also deemed “anovulatory” by BBT quantitative interpretation (no high-BBT phase). However, only seven cycles were “potentially fertile,” including a longer-than-normal or “oligomenorrheic” (11) 37-day cycle. Consistent with the findings of Direito et al. (30), a large variation (22–44 days) in ovulatory cycle length was reported (27–37 days).

### 3.3. Diet

All participants reported an omnivorous diet with DQI-I scores of 67 ± 9 points [0–100 points (34)] with food records including two or more protein sources, and seven or more grain portions daily. Four registers included all food groups each day with three displaying frequent intake of nuts and/or seeds. Eight records exhibited low intake of fruits and/or vegetables, some detailing a monotonous pattern of intake. Although the DQI-I score was not associated with ovulatory status, scores >70 points were only achieved by athletes with “normal ovulatory” cycles, while those with ovulatory disturbances obtained all ≤3 of 6 points in the “empty-calorie” DQI-I component. Macronutrient and fibre intake are described in Table 5.

### 3.4. Energy availability and progesterone

**Figure 2:**
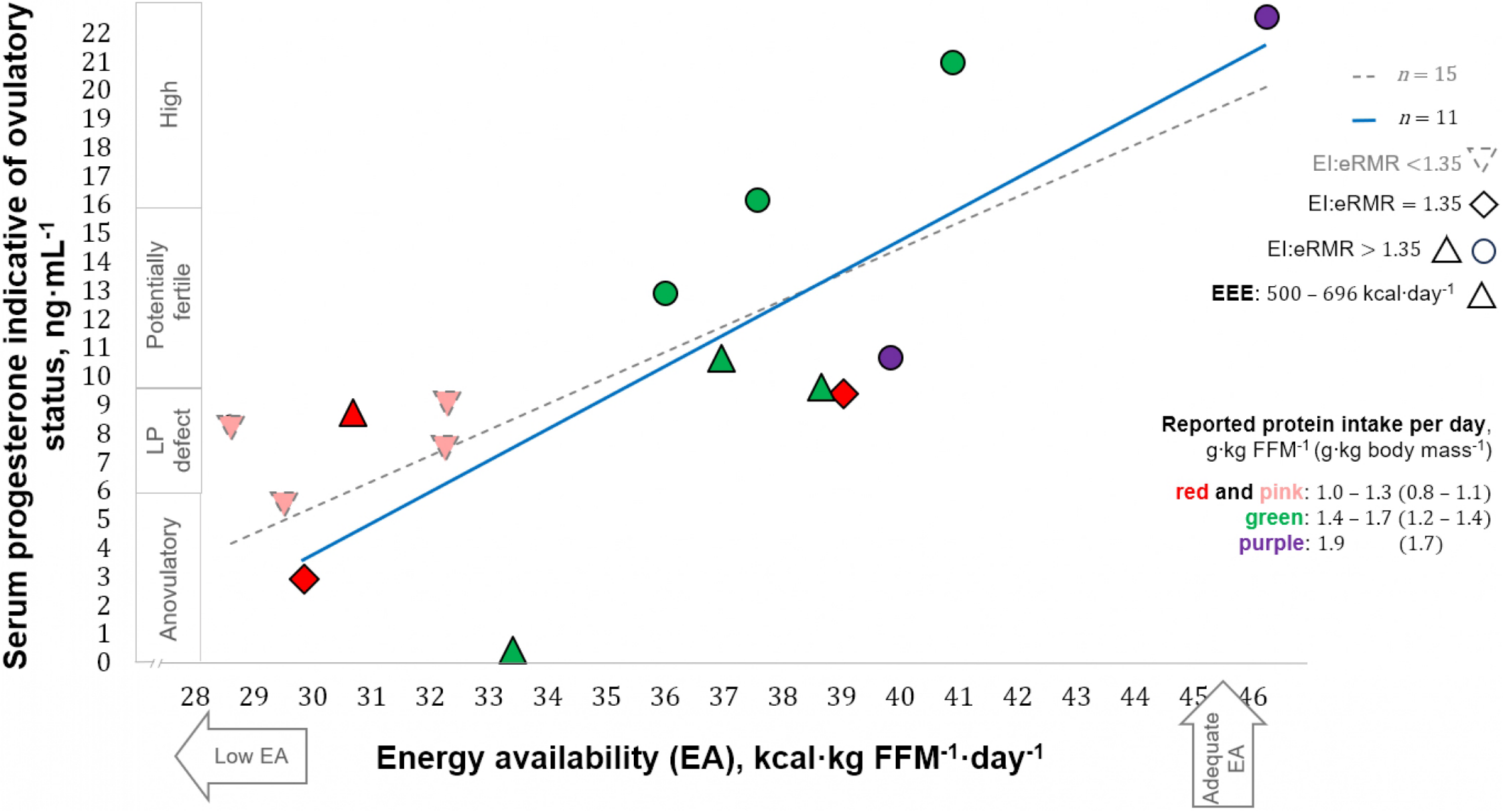
Correlation between energy availability and progesterone concentration. LP, luteal phase; FFM, fat-free mass; EI, reported energy intake; eRMR, estimated resting metabolic rate; EEE, reported exercise “training and physical activity” energy expenditure. Shapes of data points indicate EI:eRMR with triangles also showing the highest reported EEE. Colours in data points indicate reported protein intake.

**Figure 3:**
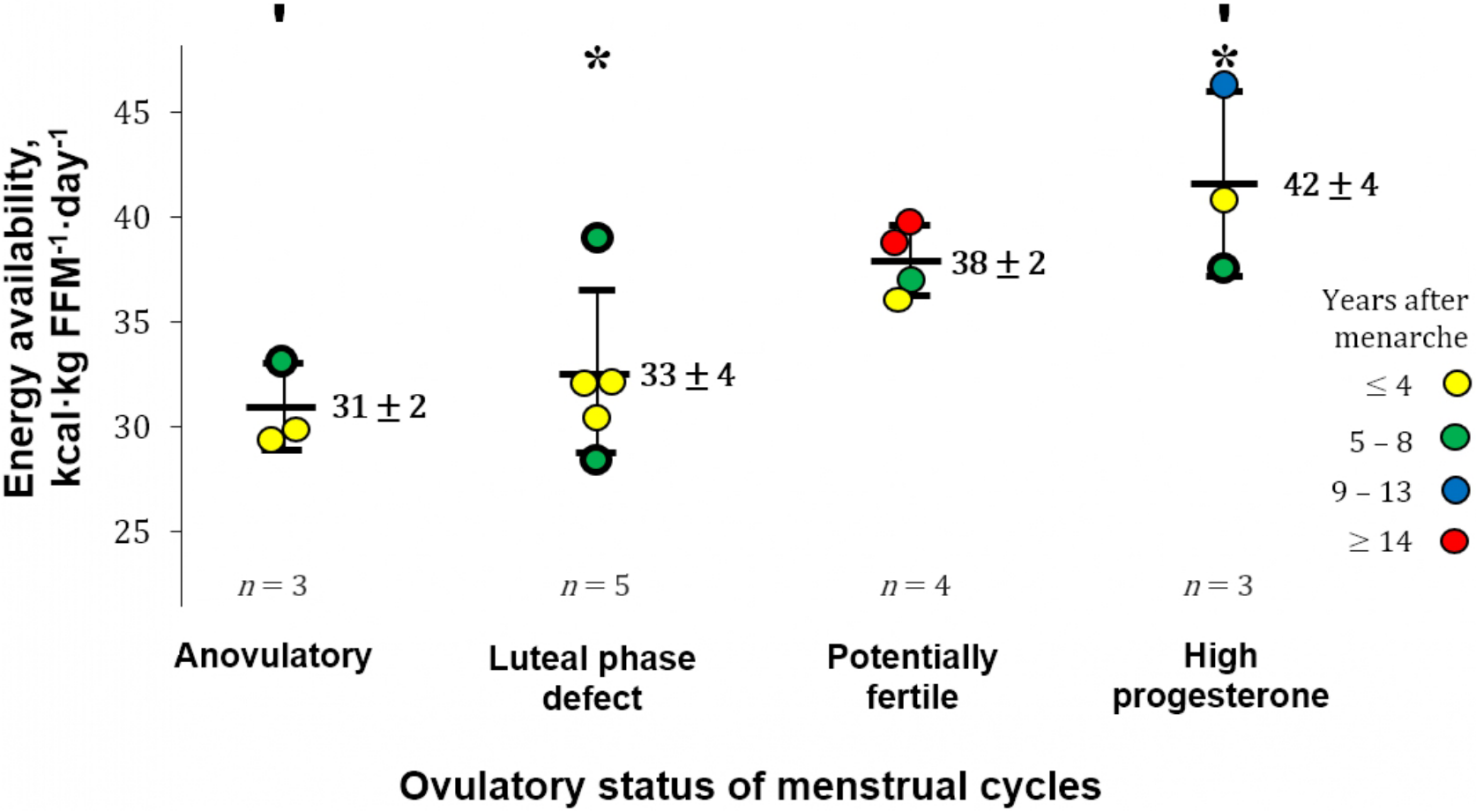
Energy availability by ovulatory status. FFM, fat-free mass; Error bars, reported mean energy availability (EA) ± *SD*. Significant differences between groups: ′*p* = 0.010 and *p = 0.014. The gynaechological age of participants is depicted in the colours of the data points. CASE HISTORIES (thick-border data points). ANOVULATORY. (1) Reported daily protein intake of 1.3 g·kg body mass^−1^ and exercise “training and physical activity” energy expenditure (EEE) of 690 kcal·day^−1^. LUTEAL PHASE DEFECT. (2) Lowest EA: The following 2 cycles were deemed ovulatory disturbed and anovulatory by quantitative interpretation of basal body temperature. (3) Highest EA: reported daily protein intake of 1.1 g·kg body mass^−1^; percentage energy intake derived from refined sugars of 14%. HIGH PROGESTERONE. (4) This elite athlete [tier 4 (50)] reported EA during the competitive season (not her highest training volume).

Estimates of EA and progesterone concentrations indicative of the ovulatory status of the studied menstrual cycles ranged from 28 to 46 kcal·kg FFM^−1^·day^−1^ and 0.41–22.51 ng·mL^−1^, respectively. EA in our participants that exhibited ovulatory disturbances (32 ± 3 kcal·kg FFM^−1^·day^−1^) appeared to be greater than in runners with more severe menstrual abnormalities, i.e., amenorrhoea [18 ± 7 kcal·kg lean body mass^−1^·day^−1^ (24)]. The ovulatory peak/mid-luteal progesterone concentrations of our participants (7.63–22.51 ng·mL^−1^) are within the range [5.39–78.5 nmol·L^−1^ (1.69–24.69 ng·mL^−1^)] quantified using similar methodology during the intermediate luteal phase defined by Anckaert et al. (51). Eleven participants reported “reduced” EA (30–45 kcal·kg FFM^−1^·day^−1^), whereas three athletes and one runner reported LEA and adequate EA, respectively. A positive correlation was observed between EA and progesterone concentration [statistics of significant correlation using our entire sample (*n* = 15) not shown]. After excluding four cases for possible underreporting or reduced EI while recording diet (EI:eRMR <1.35), a moderate correlation was observed between EA and progesterone [*r*(9) = 0.79, 95% CI: 0.37–0.94; *p* = 0.003; 1 − *β* = 0.99], with EA explaining 63% (*r^2^* = 0.63) of progesterone variance (Figure 2). Athletes with a reported EA <35 kcal·kg FFM^−1^·day^−1^ (*n* = 7) had all ovulatory disturbed cycles. In contrast, seven of eight participants with EA ≥36 kcal·kg FFM^−1^·day^−1^ exhibited progesterone indicative of “normal ovulation.” Interestingly, the exception was the racewalker that reported the lowest protein intake (1.1 g·kg body mass^−1^·day^−1^) and the highest percentage of EI derived from refined sugars (14%) in this subgroup. Linear regression analysis with EA as a predictor of peak/mid-luteal progesterone [serum progesterone concentration = 1.13 (EA) − 30.77] indicates that EA ≥36 kcal·kg FFM^−1^·day^−1^ is required to achieve normal ovulation. EA estimated during cycles with “high” progesterone was greater [*F*(3, 11) = 7.45, *p* = 0.005; 1 − *β* = 0.78] than EA during cycles with concentrations indicative of “anovulation” (*p* = 0.010) and “luteal phase deficiency” (*p* = 0.014) [mean ± SD (95% CI)]: 42 ± 4 (31–52) vs. 31 ± 2 (26–36) and 33 ± 4 (28–37) kcal·kg FFM^−1^·day^−1^ (Figure 3).

## 4. Discussion

This observational study explored the relationship between EA (estimated with field-based methodology) and the subsequent peak/mid-luteal serum progesterone concentration, which is indicative of the ovulatory status of a menstrual cycle. Our data in free-living Guatemalan competitive racewalkers and runners who prospectively recorded ≥3 cycles of normal length before this study showed a positive correlation between EA and the subsequent peak/mid-luteal progesterone concentration (Figure 2). Ovulatory disturbances (peak/mid-luteal progesterone ≤9.40 ng·mL^−1^) were observed with EA <35 kcal·kg FFM^−1^·day^−1^, and “normal ovulation” was associated with EA ≥36 kcal·kg FFM^−1^·day^−1^. Our estimates of EA (Figure 3) successfully distinguished between ovulation with “high” progesterone (>16.00 ng·mL^−1^) and both “anovulation” (≤6.00 ng·mL^−1^) and “luteal phase deficiency” (6.01–9.40 ng·mL^−1^).

Five days of LEA during the follicular phase of the menstrual cycle has been shown to disrupt the pulsatile release of LH (4). We provide further evidence of anovulation (*n* = 2) and luteal phase deficiency (*n* = 1) with progesterone concentrations during the expected peak or peak period in trained-to-highly-trained athletes who reported LEA (28–<30 kcal·kg FFM^−1^·day^−1^) during 7 consecutive days within the follicular phase of the same cycle. Insulin is critical in the control of reproduction, i.e., hyper and hypo-insulinemia are associated with disturbed gonadotropin-releasing hormone and LH pulse and release patterns (52). However, insulin declines linearly with acute EA restriction (4). Moreover, daily energy deficits (caloric restriction + exercise) of 470–813 kcal generate an incidence of ovulatory disturbances during the first menstrual cycle of 38%–42% (including 13% anovulation within the highest deficit), with incidence rates increasing and luteal phase length decreasing over three menstrual cycles (53). According to Lieberman et al. (54), the likelihood of ovulatory disturbances and oligomenorrhoea increases with a decrease in EA, with >50% probability of experiencing these disruptions with LEA, but not supporting the notion that a specific threshold of EA exists. Nevertheless, in this study (54), EEE was calculated as “total energy cost of exercise minus RMR,” while Loucks et al. (1) deducted for “non-exercise waking activity.” Furthermore, it is not clear whether the oligomenorrheic cycles reported were ovulatory disturbed which is prudent since several studies have documented extended ovulatory cycles of 36–44 days (30, 55). Intuitively, individual factors influence the onset and degree of ovulatory or menstrual disturbance during energy deficit (53). Despite these observations, it appears that acute EA restriction before (from follicular recruitment, i.e., late luteal phase of previous cycle, to LH peak) or after ovulation (from LH peak to menses) has a similar impact on the luteal phase of the actual menstrual cycle. Hence, irrespective of phase of the ovarian cycle in which an abrupt onset of short-term exercise occurred, the luteal phase was disturbed in women who did not increase their EI (56). Notably, these previous studies (4, 53, 54, 56) were conducted in habitually sedentary eumenorrheic women rather than in athletic populations. Hence, a strength of the present study is the specialised group of competitive endurance athletes recruited, although we did not control for the ovulatory status of the previous cycle. Therefore, our current investigation should be considered a pilot study to extend understanding regarding the effect of EA on the ovulatory status of free-living athletes. Future intervention studies are warranted to understand the effect of EA restriction on normal ovulation in eumenorrheic athletes and should be long-term (2–3 cycles) in design with a follow-up included (Supplementary Table S3). Given that research into how psychological stress might impact menstrual function is inconclusive (57), future studies should focus on monitoring heart rate variability (HRV) as an indicator of autonomic nervous function while being cognisant that HRV changes in response to progesterone fluctuations during normal menstrual cycles (58).

Free-living estimates of EA in highly trained and elite athletes have been considered “snapshots,” and thus not necessarily an accurate representation of long-term EA status (19). Our 7-day estimates of EA are self-reported as representative of the follicular phase, i.e., without changes in diet and exercise in the first 14 days of the studied menstrual cycle. We verified that the EA estimation period ended before the expected ovulation (Table 1) and showed that estimates of EA were related to the ovulatory status of the same menstrual cycle. This relationship is consistent with the findings of Lieberman et al. (54) that reported EA as a predictor of menstrual disturbances within the same but not the subsequent cycle. Moreover, Schliep et al. (12) showed an association between hormonal deficiencies in the follicular phase and hormonal deficiencies in the luteal phase of the same cycle. Accordingly, our observations represent an acute rather than a chronic state of EA and can only be interpreted as the relationship between EA status before ovulation and its impact on the ovulatory status within the same menstrual cycle.

Estimates of EA under free-living conditions have previously been shown to discriminate between amenorrhoea and eumenorrhea [31 ± 2 vs. 37 ± 2 kcal·kg lean body mass^−1^·day^−1^ ± SEM, respectively (6)]. However, no distinction was observed for subclinical ovulatory status as diagnosed with urinary metabolites of oestradiol and progesterone, i.e., no evidence of a statistical difference was reported regarding estimates of EA in physically active females who consistently exhibited ovulatory, disturbed, or anovulatory cycles during one to three menstrual cycles. Several methodological differences existed between our estimates of EA and those of Reed et al. (6), particularly regarding assessments of EEE, body composition, and RMR. Reed et al. (6) assessed EI over a 3-day period that was not standardised to a cycle phase, while we estimated EI and EEE simultaneously during the follicular phase with a similar timing of EA restriction in previous studies (1, 4). Given that ovulatory disturbances were expected (14, 15, 25), and since EI and expenditure are increased after ovulation (59), concomitant with slightly higher RMR during the luteal phase (60), we avoided the unfair comparison of EI between participants that would be influenced by ovulatory status. There is a lack of a single protocol for the assessment of EA in free-living situations (5). Hence, the design of our protocol did not include self-reporting a difference in diet or exercise during the first 2 weeks of the studied menstrual cycle. However, during the first two cycles studied, we recognised the importance of exhibiting EA estimates representative of the follicular phase. Our free-living estimates of EA were correlated with peak/mid-luteal progesterone concentration and distinguished between ovulatory status. To our knowledge, this is the first report of a significant association between EA and peak/mid-luteal progesterone concentration in free-living competitive endurance athletes.

Monitoring the menstrual cycle of athletes in a free-living situation is challenging. In the context of “normal ovulatory” or “fertile” menstrual cycles, progesterone remains elevated from day 10 to day 5 prior to menstruation (13). Progesterone was assessed 4 days before the last day in two cycles, near the midpoint of their post-ovulatory phases. One of these cycles exhibited a 9-day (short) luteal phase with progesterone concentration quantified 6 days after the day of LH peak. However, if the assessment was planned 9 days after the LH peak [within the peak period in “normal” cycles (10)], we would have failed to document peak progesterone with a quantification scheduled 1 day before the last of this cycle. Thus, practitioners or researchers that use our protocol to control for cycle phase or document progesterone concentration during the peak period in athletes with expected ovulatory disturbances should consider that assessments are based on “normal” cycles. In the present study, as ovulatory disturbances became severe, it was more difficult to quantify progesterone within the peak period, which in a normal cycle is from about 2 days before until 2 days after the middle of the luteal phase.

Prior evidence suggests that normal-length menstrual cycles may mask ovulatory disturbances (15, 25). The prevalence rate of ovulatory disturbances in our cohort of athletes was 53% which falls within the range (29%–79%) that was previously reported in active females (14, 15, 25). Our data also suggest that monitoring menstrual cycle length effectively detects disturbances until deemed clinically evident with substantial delay or absence of menstruation. However, recent evidence indicates that menstrual cycle monitoring is not widespread, with only 54% of elite and highly trained athletes tracking their cycles (61). Hence, rather than waiting until the period is absent, practitioners are encouraged to take a proactive approach to detecting LEA at an early stage as consequences of RED-S such as anaemia, decreased muscle strength and glycogen stores, vulnerability to illness, and stress fractures can be detrimental to health and performance (18). Thus, in practical terms, practitioners supporting athletes vulnerable to RED-S may attempt to monitor the ovulatory status of those individuals not using hormonal contraception because hormonal contraceptives will often prevent ovulation. Based on our LH detection analysis, it was possible to record short luteal phases [positive urinary LH detection ≤10 days before the last day of the cycle, defining a luteal length of <10 days (12)], although this activity required continued searching from the expected timing of LH peak until almost the end of the menstrual cycle. Whereas Park et al. (55) documented anovulation in 4.7% of cycles with LH surges, we observed three false LH “positives for ovulation” with a level of urinary detection of ≥30 mIU·mL^−1^ (Table 1 and Supplementary Table S2). Furthermore, we documented normal-length luteal phases with peak progesterone indicative of “luteal phase deficiency.” Therefore, monitoring the timing of “ovulation” is not sufficient in athletic populations, where the assessment of hormonal adequacy in the luteal phase becomes important.

Establishing comprehensive guidelines for EA restriction to achieve body composition goals in female athletes warrants further investigation, alongside practical means of monitoring ovulatory status. In female athletes, a decline in body mass of 0.7% per week appears commensurate with resistance training goals (62). However, recommendations for EA restriction without disturbing the ovulatory status to the extent of impairing long-term health and performance remain undetermined. While fertility might not be a concern for many athletes, ovulatory cycles also have a role in bone health (10, 15). Indeed, a lower than expected bone mineral density (z-score < 0) has been associated with LEA (63). In addition, athletes should be aware that LEA impairs muscle protein synthesis (64) and that failure of progesterone to rise adequately during the menstrual cycle is not only associated with LEA but also with impaired athletic performance (65), i.e., when athletes fail to increase EI with increased training loads, ovulatory disturbances are induced and performance is impaired (26). Hence, the practitioner may monitor progesterone during the peak period as a tool to confirm an EA status that is sufficient to maintain normal ovulatory function in naturally menstruating athletes, instead of the time-consuming estimation of EA. However, there could be exceptions in terms of ovulatory or menstrual disturbances without obvious indications of chronic energy deficiency, such as in polycystic ovary syndrome (66). Moreover, the quantitative interpretation of carefully documented BBT during menstrual cycles may be considered equally promising as a non-invasive and low-cost screening tool to assess ovulatory status. Our BBT data support the use of BBT quantitative interpretation to indicate a peak, mid-luteal, or progesterone concentration at the expected peak (Castellanos-Mendoza et al., unpublished observations), although further validation in the context of preventing LEA is needed. Given that some female athletes use hormonal contraception methods that may mask LEA (67) as users may interpret withdrawal bleeding as a “period” when they may otherwise have menstrual disturbances, it remains relevant to establish the minimum EA required to maintain normal ovulation to guide the prescription of energy restriction. Since access to gold standard methods is limited, a parallel estimation of EA with accessible methodology and an indication of the equivalent energy deficit is useful for practitioners. In Supplementary Tables S2, S3, we provide our feasibility analysis and suggestions for future research.

The challenges of estimating EA in a field setting are well recognised (5, 67); hence, care was taken in the present study to minimise the chance of possible errors, with special emphasis on the estimation of EI and the total energy cost of training and physical activity (Methods and Supplementary Table S1). While adding food images to food diaries may reduce the margin of error (68), participant motivation and “attitude to food” likely influence the validity of self-reported EI (69). In terms of energy balance (EB) expressed as percentage of total energy expenditure (TEE), the reported mean error of EI by weighed diet records without food images was −34% to −1% in female athletes with estimated TEE by doubly labelled water (DLW) (33, 69). The highest difference was observed in runners that were required to self-report dietary intake over 3 weeks, and light-weight rowers. In contrast, the smallest difference was documented when dietitians weighed food portions during meals, with snacks and sports drinks considered as the source of individual error [−18.4% to +19.2% (70)]. Nevertheless, most previous studies included a shorter EI assessment period than estimation of TEE, and no study controlled EEE or derived “EEE + NEAT (non-exercise activity thermogenesis)” from TEE and RMR as in Silva et al. (71). Moreover, the degree of error explained by undereating, underreporting, and/or dietary analysis drawbacks remains unknown (69). Although the limitations of using METs to estimate individual energy cost of exercise are recognised (37, 38), 11 of 25 studies reviewed by Burke et al. (5) used METs as an independent metric of self-reported EEE estimation. To our knowledge, no validation study has been conducted to address the error in using METs to estimate EEE from training and physical activity diaries in female athletes. TEE determined by DLW ranged from 2,350 to 3,735 kcal·day^−1^ in free-living female runners (72, 73) that on average were older, taller, of greater body mass, and with higher training volume than the participants of this study. Interestingly, the estimated TEE [eRMR·1.3 (1.33 for adolescents) + EEE] with our data was 1,821–2,453 kcal·day^−1^, with EB remarkably similar to previous reports (−11% ± 12%; −30% to +17%).

To our knowledge, this study is novel in suggesting that a threshold for EA is associated with ovulatory disturbances and in highlighting the impact of EA on the serum peak/mid-luteal progesterone concentration. A graphical summary of the short-term effects of LEA on hormones [Figure 3 in Areta et al. (74)] shows the lack of evidence in terms of peak progesterone concentration. Linear regression analysis suggests that EA = 35 kcal·kg FFM^−1^·day^−1^ reflects ovulatory disturbances, but this EA value was not reported. Due to our study limitations, including lack of precision in field-based methods, lack of control of variables known to alter the menstrual cycle in a free-living setting, a small sample size, and use of two laboratories to quantify progesterone, we report the threshold EA between ovulatory disturbances and “normal ovulation” with a gap representing our observations without statistical analysis. These EA thresholds for ovulatory disturbances (EA <35 kcal·kg FFM^−1^·day^−1^) and “normal ovulation” (EA ≥36 kcal·kg FFM^−1^·day^−1^) are based on the participants studied with our methodology. Highlighting the pilot nature of this study, we speculate that other methods to estimate EA and participant restriction by training and performance classification, or years after menarche (y.a.m.) [2–4 y.a.m. “adolescents” vs. > 14 y.a.m. “mature women” (75)] likely impact the threshold EA for ovulatory disturbances. While our analysis is limited to carbohydrate intake per FFM (Table 5) as we did not assess the intensity or metabolic effect of EEE, it remains unknown if carbohydrate availability [intake minus oxidation during exercise (76)] has a greater impact than EA on the ovulatory status.

Two additional limitations are associated with this study. First, recreationally active (tier 1) and world-class (tier 5) athletes were not represented, while elite (tier 4) athletes (50) were underrepresented and most participants (87%) were between 2 and 9 years post-menarche. Consequently, our findings can only reliably be extrapolated to trained and highly trained (tier 2–3) female athletes aged 14–23 years given that most participants were within this age range except for two aged 27 and 41 years. Second, we did not conduct interviews or questionnaires to investigate eating behaviour to objectively verify “restrictive eaters” and discern whether the low EI was due to “consciously eating less while keeping a detailed register of food intake” rather than “underreporting.” Nevertheless, we excluded athletes with EI:eRMR <1.35 to formulate our conclusion.

## 5. Conclusions

We conclude that EA during the follicular phase of the menstrual cycle impacts the ovulatory status of the same cycle in competitive racewalkers and runners. Our free-living estimates of EA <35 and ≥36 kcal·kg FFM^−1^·day^−1^ are associated with subsequent progesterone concentrations indicative of ovulatory disturbances and normal ovulation, respectively. Further research is warranted to elucidate the threshold EA associated with ovulatory disturbances in athletes and develop non-invasive means of monitoring ovulatory status in trained individuals.

## Data Availability

Due to participant confidentiality and privacy, an unidentifiable data set supporting the conclusions of this article is only available upon request to be directed to the corresponding author.

## References

[1] Loucks AB Verdun M Heath EM. Low energy availability, not stress of exercise, alters LH pulsatility in exercising women. J Appl Physiol*.* (1998) 84(1):37–46. 10.1152/jappl.1998.84.1.37 9451615

[2] Mulligan K Butterfield G. Discrepancies between energy intake and expenditure in physically active women. Br J Nutr*.* (1990) 64(1):23–36. 10.1079/BJN19900006 2400763

[3] Ihle R Loucks AB. Dose–response relationships between energy availability and bone turnover in young exercising women. J Bone Miner Res*.* (2004) 19(8):1231–40. 10.1359/JBMR.040410 15231009

[4] Loucks AB Thuma JR. Luteinizing hormone pulsatility is disrupted at a threshold of energy availability in regularly menstruating women. J Clin Endocrinol Metab*.* (2003) 88(1):297–311. 10.1210/jc.2002-020369 12519869

[5] Burke LM Lundy B Fahrenholtz IL Melin AK. Pitfalls of conducting and interpreting estimates of energy availability in free-living athletes. Int J Sport Nutr Exerc Metab*.* (2018) 28(4):350–63. 10.1123/ijsnem.2018-0142 30029584

[6] Reed JL De Souza MJ Mallinson RJ Scheid JL Williams NI. Energy availability discriminates clinical menstrual status in exercising women. J Int Soc Sports Nutr*.* (2015) 12:11. 10.1186/s12970-015-0072-0 25722661 PMC4342163

[7] Reed BG Carr BR. (2015). *The normal menstrual cycle and the control of ovulation*. [updated May 22, 2015]. In: De Groot L.J. Chrousos G. Dungan K., editors. Endotext. South Dartmouth, MA: MDText.com, Inc. (2000). p. 14–23. Available at: https://www.ncbi.nlm.nih.gov/books/NBK279054 (Accessed January 2017).

[8] Baird DT Bäckström T McNeilly AS Smith SK Wathen CG. Effect of enucleation of the corpus luteum at different stages of the luteal phase of the human menstrual cycle on subsequent follicular development. J Reprod Fertil*.* (1984) 70:615–24. 10.1530/jrf.0.0700615 6422035

[9] Prior JC Naess M Langhammer A Forsmo S. Ovulation prevalence in women with spontaneous normal-length menstrual cycles—a population-based cohort from HUNT3, Norway. PLoS One. (2015) 10(8):e0134473. 10.1371/journal.pone.0134473 26291617 PMC4546331

[10] Niethammer B Körner C Schmidmayr M Luppa PB Seifert-Klauss VR. Non-reproductive effects of anovulation: bone metabolism in the luteal phase of premenopausal women differs between ovulatory and anovulatory cycles. Geburtshilfe Frauenheilkd. (2015) 75(12):1250–7. 10.1055/s-0035-1558298 26726266 PMC4686368

[11] Elliott-Sale KJ Minahan CL Janse de Jonge XAK Ackerman KE Sipilä S Constantini NW Methodological considerations for studies in sport and exercise science with women as participants: a working guide for standards of practice for research on women. Sports Med*.* (2021) 51:843–61. 10.1007/s40279-021-01435-8 33725341 PMC8053180

[12] Schliep KC Mumford SL Hammoud AO Stanford JB Kissell KA Sjaarda LA Luteal phase deficiency in regularly menstruating women: prevalence and overlap in identification based on clinical and biochemical diagnostic criteria. J Clin Endocrinol Metab*.* (2014) 99(6):E1007–14. 10.1210/jc.2013-3534 24606080 PMC4037737

[13] Hull MG Savage PE Bromham DR Ismail AA Morris AF. The value of a single serum progesterone measurement in the midluteal phase as a criterion of a potentially fertile cycle (“ovulation”) derived from treated and untreated conception cycles. Fertil Steril*.* (1982) 37(3):355–60. 10.1016/s0015-0282(16)46095-4 7060786

[14] De Souza MJ Miller BE Sequenzia LC Luciano AA Ulreich S Stier S Bone health is not affected by luteal phase abnormalities and decreased ovarian progesterone production in female runners. J Clin Endocrinol Metab*.* (1997) 82(9):2867–76. 10.1210/jcem.82.9.4201 9284712

[15] Prior JC Vigna YM Schechter MT Burgess AE. Spinal bone loss and ovulatory disturbances. N Engl J Med*.* (1990) 323(18):1221–7. 10.1056/NEJM199011013231801 2215605

[16] Li D Hitchcock CL Barr SI Yu T Prior JC. Negative spinal bone mineral density changes and subclinical ovulatory disturbances—prospective data in healthy premenopausal women with regular menstrual cycles. Epidemiol Rev*.* (2014) 36:137–47. 10.1093/epirev/mxt012 24275546

[17] Nattiv A Loucks AB Manore MM Sanborn CF Sundgot-Borgen J Warren MP American College of sports medicine position stand. The female athlete triad. Med Sci Sports Exerc*.* (2007) 39(10):1867–82. 10.1249/mss.0b013e318149f111 17909417

[18] Mountjoy M Ackerman KE Bailey DM Burke LM Constantini N Hackney AC 2023 International Olympic Committee’s (IOC) consensus statement on relative energy deficiency in sport (REDs). B J Sports Med*.* (2023) 57(17):1073–97. 10.1136/bjsports-2023-106994 37752011

[19] Melin A Tornberg AB Skouby S Møller S Sundgot-Borgen J Faber J Energy availability and the female athlete triad in elite endurance athletes. Scand J Med Sci Sports. (2015) 25(5):610–22. 10.1111/sms.12261 24888644

[20] Thein-Nissenbaum JM Rauh MJ Carr KE Loud KJ McGuine TA. Menstrual irregularity and musculoskeletal injury in female high school athletes. J Athl Train*.* (2012) 47(1):74–82. 10.4085/1062-6050-47.1.74 22488233 PMC3418118

[21] Hutson MJ O’Donnell E Brooke-Wavell K Sale C Blagrove RC. Effects of low energy availability on bone health in endurance athletes and high-impact exercise as a potential countermeasure: a narrative review. Sports Med*.* (2021) 51(3):391–403. 10.1007/s40279-020-01396-4 33346900 PMC7900047

[22] Heikura IA Uusitalo AL Stellingwerff T Bergland D Mero AA Burke LM. Low energy availability is difficult to assess but outcomes have large impact on bone injury rates in elite distance athletes. Int J Sport Nutr Exerc Metab*.* (2018) 28(4):403–11. 10.1123/ijsnem.2017-0313 29252050

[23] Loucks AB. Low energy availability in the marathon and other endurance sports. Sports Med*.* (2007) 37:348–52. 10.2165/00007256-200737040-00019 17465605

[24] Schaal K Van Loan MD Casazza GA. Reduced catecholamine response to exercise in amenorrheic athletes. Med Sci Sports Exerc*.* (2011) 43(1):34–43. 10.1249/MSS.0b013e3181e91ece 20508538

[25] De Souza MJ Miller BE Loucks AB Luciano AA Pescatello LS Campbell CG High frequency of luteal phase deficiency and anovulation in recreational women runners: blunted elevation in follicle-stimulating hormone observed during luteal-follicular transition. J Clin Endocrinol Metab*.* (1998) 83(12):4220–32. 10.1210/jcem.83.12.5334 9851755

[26] Schaal K VanLoan MD Hausswirth C Casazza GA. Decreased energy availability during training overload is associated with non-functional overreaching and suppressed ovarian function in female runners. Appl Physiol Nutr Metab*.* (2021) 46(10):1179–88. 10.1139/apnm-2020-0880 33651630

[27] Melin A Tornberg AB Skouby S Faber J Ritz C Sjödin A The LEAF questionnaire: a screening tool for the identification of female athletes at risk for the female athlete triad. Br J Sports Med*.* (2014) 48(7):540–5. 10.1136/bjsports-2013-093240 24563388

[28] Pallone SR Bergus GR. Fertility awareness-based methods: another option for family planning. J Am Board Fam Med*.* (2009) 22(2):147–57. 10.3122/jabfm.2009.02.080038 19264938

[29] Kesner JS Knecht EA Krieg EF Jr Wilcox AJ O’Connor JF. Detecting pre-ovulatory luteinizing hormone surges in urine. Hum Reprod*.* (1998) 13(1):15–21. 10.1093/humrep/13.1.15 9512221

[30] Direito A Bailly S Mariani A Ecochard R. Relationships between the luteinizing hormone surge and other characteristics of the menstrual cycle in normally ovulating women. Fertil Steril*.* (2013) 99(1):279–85.e3. 10.1016/j.fertnstert.2012.08.047 22999798

[31] Behre HM Kuhlage J Gassner C Sonntag B Schem C Schneider HP Prediction of ovulation by urinary hormone measurements with the home use ClearPlan® fertility monitor: comparison with transvaginal ultrasound scans and serum hormone measurements. Hum Reprod*.* (2000) 15(12):2478–82. 10.1093/humrep/15.12.2478 11098014

[32] Centre for Menstrual Cycle and Ovulation Research ‘CeMCOR’, University of British Columbia. (n.d.) *The Quantitative Basal Temperature method for determining ovulation and luteal phase length*. Available at: http://www.cemcor.ca/files/uploads/QBT_instructions.pdf (Accessed January 2018).

[33] Capling L Beck KL Gifford JA Slater G Flood VM O’Connor H. Validity of dietary assessment in athletes: a systematic review. Nutrients. (2017) 9(12):1313. 10.3390/nu9121313 29207495 PMC5748763

[34] INDDEX Project. (2018). *Diet Quality Index—International (DQI-I)*. Data4diets: building blocks for diet-related food security analysis. Tufts University, Boston, MA. Available at: https://inddex.nutrition.tufts.edu/data4diets/indicator/diet-quality-index-international-dqi-i?back=/data4diets/indicators (Accessed November 6, 2022).

[35] Harris JA Benedict FG. A biometric study of human basal metabolism. Proc Natl Acad Sci U S A*.* (1918) 4(12):370–3. 10.1073/pnas.4.12.370 16576330 PMC1091498

[36] Schofield WN. Predicting basal metabolic rate, new standards and review of previous work. Hum Nutri Clin Nutr*.* (1985) 39(Suppl 1):5–41. PMID: . 4044297

[37] Ainsworth BE Haskell WL Herrmann SD Meckes N Bassett DR Jr Tudor-Locke C 2011 Compendium of physical activities: a second update of codes and MET values. Med Sci Sports Exerc*.* (2011) 43(8):1575–81. 10.1249/MSS.0b013e31821ece12 21681120

[38] Butte NF Watson KB Ridley K Zakeri IF McMurray RG Pfeiffer KA A youth compendium of physical activities: activity codes and metabolic intensities. Med Sci Sports Exerc*.* (2018) 50(2):246–56. 10.1249/MSS.0000000000001430 28938248 PMC5768467

[39] Yuhasz MS. Physical fitness manual. London, Ontario: University of Western Ontario (1974).

[40] Wong JE Poh BK Nik-Shanita S Izham MM Chan KQ Tai MD Predicting basal metabolic rates in Malaysian adult elite athletes. Singapore Med J*.* (2012) 53(11):744–9. PMID: . 23192502

[41] Carlsohn A Scharhag-Rosenberger F Cassel M Mayer F. Resting metabolic rate in elite rowers and canoeists: difference between indirect calorimetry and prediction. Ann Nutr Metab*.* (2011) 58(3):239–44. 10.1159/000330119 21811063

[42] Torún B Menchú MT Elias LG. Recomendaciones dietéticas diarias del INCAP. Edición XLV aniversario. Publicación INCAP ME/057. Guatemala: INCAP-OPS (1994).

[43] WHO. Energy and protein requirements. Report of a joint FAO/WHO/UNU expert consultation. World Health Organ Tech Rep Ser*.* (1985) 724:1–206. PMID: . 3937340

[44] Guebels CP Kam LC Maddalozzo GF Manore MM. Active women before/after an intervention designed to restore menstrual function: resting metabolic rate and comparison of four methods to quantify energy expenditure and energy availability. Int J Sport Nutr Exerc Metab*.* (2014) 24(1):37–46. 10.1123/ijsnem.2012-0165 23918617

[45] Hagber JM Coyle EF. Physiologic comparison of competitive race-walking and running. Int J Sports Med*.* (1984) 5:74–7. 10.1055/s-2008-1025883 6715100

[46] Mora-Rodriguez R Ortega JF Hamouti N. In a hot-dry environment racewalking increases the risk of hyperthermia in comparison to when running at a similar velocity. Eur J Appl Physiol*.* (2011) 111(6):1073–80. 10.1007/s00421-010-1733-y 21113615

[47] White CP Hitchcock CL Vigna YM Prior JC. Fluid retention over the menstrual cycle: 1-year data from the prospective ovulation cohort. Obstet Gynecol Int*.* (2011) 2011:138451. 10.1155/2011/138451 21845193 PMC3154522

[48] Goldberg GR Black AE Jebb SA Cole TJ Murgatroyd PR Coward WA Critical evaluation of energy intake data using fundamental principles of energy physiology: 1. Derivation of cut-off limits to identify under-recording. Eur J Clin Nutr*.* (1991) 45(12):569–81. PMID: . 1810719

[49] Faul F Erdfelder E Buchner A Lang AG. Statistical power analyses using G*power 3.1: tests for correlation and regression analyses. Behav Res Methods. (2009) 41(4):1149–60. 10.3758/BRM.41.4.1149 19897823

[50] McKay AK Stellingwerff T Smith ES Martin DT Mujika I Goosey-Tolfrey VL Defining training and performance caliber: a participant classification framework. Int J Sports Physiol Perform*.* (2022) 17(2), 317–31. 10.1123/ijspp.2021-0451 34965513

[51] Anckaert E Jank A Petzold J Rohsmann F Paris R Renggli M Extensive monitoring of the natural menstrual cycle using the serum biomarkers estradiol, luteinizing hormone and progesterone. Pract Lab Med. (2021) 25:e00211. 10.1016/j.plabm.2021.e00211 33869706 PMC8042396

[52] Sliwowska JH Fergani C Gawałek M Skowronska B Fichna P Lehman MN. Insulin: its role in the central control of reproduction. Physiol Behav*.* (2014) 133:197–206. 10.1016/j.physbeh.2014.05.021 24874777 PMC4084551

[53] Williams NI Leidy HJ Hill BR Lieberman JL Legro RS De Souza MJ. Magnitude of daily energy deficit predicts frequency but not severity of menstrual disturbances associated with exercise and caloric restriction. Am J Physiol Endocrinol Metab*.* (2015) 308(1):E29–39. 10.1152/ajpendo.00386.2013 25352438 PMC4281686

[54] Lieberman JL De Souza MJ Wagstaff DA Williams NI. Menstrual disruption with exercise is not linked to an energy availability threshold. Med Sci Sports Exerc*.* (2018) 50(3):551–61. 10.1249/MSS.0000000000001451 29023359 PMC5820163

[55] Park SJ Goldsmith LT Skurnick JH Wojtczuk A Weiss G. Characteristics of the urinary luteinizing hormone surge in young ovulatory women. Fertil Steril*.* (2007) 88(3):684–90. 10.1016/j.fertnstert.2007.01.045 17434509

[56] Williams NI Bullen BA McArthur JW Skrinar GS Turnbull BA. Effects of short-term strenuous endurance exercise upon corpus luteum function. Med Sci Sports Exerc*.* (1999) 31(7):949–58. 10.1097/00005768-199907000-00006 10416555

[57] Loucks AB Redman LM. The effect of stress on menstrual function. Trends Endocrinol Metab*.* (2004) 15(10):466–71. 10.1016/j.tem.2004.10.005 15541645

[58] Schmalenberger KM Eisenlohr-Moul TA Jarczok MN Eckstein M Schneider E Brenner IG Menstrual cycle changes in vagally-mediated heart rate variability are associated with progesterone: evidence from two within-person studies. J Clin Med*.* (2020) 9(3):617. 10.3390/jcm9030617 32106458 PMC7141121

[59] Davidsen L Vistisen B Astrup A. Impact of the menstrual cycle on determinants of energy balance: a putative role in weight loss attempts. Int J Obes (Lond)*.* (2007) 31:1777–85. 10.1038/sj.ijo.0803699 17684511

[60] Solomon SJ Kurzer MS Calloway DH. Menstrual cycle and basal metabolic rate in women. Am J Clin Nutr*.* (1982) 36(4):611–6. 10.1093/ajcn/36.4.611 7124662

[61] Heather AK Thorpe H Ogilvie M Sims ST Beable S Milsom S Biological and socio-cultural factors have the potential to influence the health and performance of elite female athletes: a cross sectional survey of 219 elite female athletes in Aotearoa New Zealand. Front Sports Act Living. (2021) 3:601420. 10.3389/fspor.2021.601420 33681758 PMC7932044

[62] Garthe I Raastad T Refsnes PE Koivisto A Sundgot-Borgen J. Effect of two different weight-loss rates on body composition and strength and power-related performance in elite athletes. Int J Sport Nutr Exerc Metab*.* (2011) 21(2):97–104. 10.1123/ijsnem.21.2.97 21558571

[63] Viner RT Harris M Berning JR Meyer NL. Energy availability and dietary patterns of adult male and female competitive cyclists with lower than expected bone mineral density. Int J Sport Nutr Exerc Metab*.* (2015) 25 (6):594–602. 10.1123/ijsnem.2015-0073 26131616

[64] Oxfeldt M Phillips SM Andersen OE Johansen FT Bangshaab M Risikesan J Low energy availability reduces myofibrillar and sarcoplasmic muscle protein synthesis in trained females. J Physiol*.* (2023) 601(16):3481–97. 10.1113/JP284967 37329147

[65] Vanheest JL Rodgers CD Mahoney CE De Souza MJ. Ovarian suppression impairs sport performance in junior elite female swimmers. Med Sci Sports Exerc*.* (2014) 46(1):156–66. 10.1249/MSS.0b013e3182a32b72 23846160

[66] Hagmar M Berglund B Brismar K Hirschberg AL. Hyperandrogenism may explain reproductive dysfunction in Olympic athletes. Med Sci Sports Exerc*.* (2009) 41(6):1241–8. 10.1249/MSS.0b013e318195a21a 19461542

[67] Heikura IA Stellingwerff T Areta JL. Low energy availability in female athletes: from the lab to the field. Eur J Sport Sci*.* (2022) 22(5):709–19. 10.1080/17461391.2021.1915391 33832385

[68] Pettitt C Liu J Kwasnicki R Yang G Preston T Frost G. A pilot study to determine whether using a lightweight, wearable micro-camera improves dietary assessment accuracy and offers information on macronutrients and eating rate. Br J Nutr*.* (2016) 115(1):160–7. 10.1017/S0007114515004262 26537614

[69] Hill RJ Davies PS. The validity of self-reported energy intake as determined using the doubly labelled water technique. Br J Nutr*.* (2001) 85(4):415–30. 10.1079/bjn2000281 11348556

[70] Sjödin AM Andersson AB Högberg JM Westerterp KR. Energy balance in cross-country skiers: a study using doubly labeled water. Med Sci Sports Exerc*.* (1994) 26(6):720–4. 10.1249/00005768-199406000-00011 8052113

[71] Silva AM Matias CN Santos DA Thomas D Bosy-Westphal A Müller MJ Compensatory changes in energy balance regulation over one athletic season. Med Sci Sports Exerc*.* (2017) 49(6):1229–35. 10.1249/MSS.0000000000001216 28121799

[72] Edwards JE Lindeman AK Mikesky AE Stager JM. Energy balance in highly trained female endurance runners. Med Sci Sports Exerc. (1993) 25 (12):1398–1404. PMID: . 8107549

[73] Schulz LO Alger S Harper I Wilmore JH Ravussin E. Energy expenditure of elite female runners measured by respiratory chamber and doubly labeled water. J Appl Physiol (1985). (1992) 72(1):23–8. 10.1152/jappl.1992.72.1.23 1537719

[74] Areta JL Taylor HL Koehler K. Low energy availability: history, definition and evidence of its endocrine, metabolic and physiological effects in prospective studies in females and males. Eur J Appl Physiol*.* (2021) 121:1–21 10.1007/s00421-020-04516-0 33095376 PMC7815551

[75] Loucks AB. The response of luteinizing hormone pulsatility to 5 days of low energy availability disappears by 14 years of gynecological age. J Clin Endocrinol Metab. (2006) 91(8):3158–64. 10.1210/jc.2006-0570 16720651

[76] Loucks AB. Energy balance and body composition in sports and exercise. J Sports Sci*.* (2004) 22(1):1–14. 10.1080/0264041031000140518 14974441

[77] Castellanos-Mendoza MC. Energy availability estimated in free-living conditions positively correlates with peak progesterone concentration in the menstrual cycle of race walkers and runners not using hormonal contraception. In: abstracts from the December 2019 international sport + exercise nutrition conference in Newcastle upon Tyne. Int J Sport Nutr Exerc Metab. (2020) 30(S1):1–14. 10.1123/ijsnem.2020-006531945739

