# Supplementary material for: Free-living competitive racewalkers and runners with energy availability estimates of <35 kcal·kg fat-free mass−1·day−1 exhibit peak serum progesterone concentrations indicative of ovulatory disturbances: a pilot study
Source: Front Sports Act Living. 2023 Nov 17;5:1279534. doi: 10.3389/fspor.2023.1279534 (PMC10690956; doi:10.3389/fspor.2023.1279534)
Supplement: Supplementary file 1 [file Datasheet1.pdf]

Supplementary Material

TABLE S1| Details of data collection for energy availability estimation.

**Dietary energy intake**<br>'EI'<br><br>Weighed and photographed food diary	*Motivation and instruction*<br>• Importance of the study: eat as usual and report accurately<br>• Written guidelines with an example of 1 day filled in the form, including individual foods, sports drinks, and family recipes<br>• Demonstration of how to: 1) fill in the food diary form, 2) use the kitchen scale<br><br>*Form*<br>• Portion size: measuring cups/spoons or small/large/etc. plus the number of units; volume in mL (sports drinks, alcohol)<br>• Description: food type, ingredients, cooking methods, added fat and sugar, brand<br>• Weight, grams: portion served, uneaten foods/waste, plate/glass if needed to be subtracted (i.e., unable to tare, such as if food/drink is served already)<br>• Family meal recipes for *ratatouille*-type dishes: weight of ingredients and proportion eaten by the participant<br>• Processed food packages: retaining or photographing nutritional data<br><br>*Equipment*<br>• Kitchen scales: Guzzini 1808104 and Watshome PT-808 (5 kg capacity, 1 g precision, portable and digital), both registering the same weight when objects are placed centred<br>• Personal smartphone camera: photographic evidence of meals and snacks
**Exercise (training and physical activity) energy expenditure**<br>'EEE'<br><br>Training and physical activity diary	*Instruction*<br>• Written guidelines with examples of several days filled in the form, including all types of training, household, and leisure/social physical activities<br>• List of activities that needed to be reported in the instruction sheet<br>• Explanation of how to fill the form to 1) athletes, 2) research assistants<br><br>*Form*<br>• Description of the workout: activity, exercise; number and length of laps, etc.<br>• Running or racewalking 1) surface: unpaved/paved road, dirt/synthetic track, etc., and 2) inclination: % of the route up/downhill, slight/moderate/steep hill<br>• Distance and time of racewalking or running indicated with the highest possible precision (walking <3.5 METs, i.e., recovery, excluded)<br>• Duration of each specific training activity: measured with watch or chronometer of athlete or observer (recovery time excluded)<br>• Heart rate upon finishing tasks: athlete counted heartbeats with her hand on her chest for 10 seconds or as registered in her watch
Anthropometry to estimate basal metabolic rate	*Equipment to measure body mass and height using ISAK protocol*<br>• Person scale: Tanita BC-313 (50 g accuracy)<br>• Stadiometer: Seca 213 (0.1 cm precision)
**Fat-free mass**<br>'FFM'	*Anthropometric measurements for estimation of body fat-free mass*<br>• Skinfolds: measured in duplicate (or triplicate if necessary) by a certified anthropometry practitioner (ISAK Level 1) with Slim Guide calliper<br>• Body fat %: Excel® template received at the ISAK Level 1 certification course in Morelia, Mexico, 2015, that requires 6 skinfolds (triceps, subscapular, supraspinale, abdominal, thigh, calf) to estimate as per equation in Yuhasz (1974)

METs: Metabolic equivalent of tasks; ISAK: International Society for the Advancement of Kinanthropometry

TABLE S2 │ Feasibility analysis.

**Recruitment, enrollment, and starting the study**	• *Finding potential participants*: easy in a culture in which females barely use hormonal contraception<br>• Delay *from screening to enrollment*: after scoring the screening questionnaire and explaining the study, most (82%) eligible athletes chose to enroll. Racewalkers and runners not yet competing internationally (tier 2, McKay et al., 2022) had little/no medical/nutritional support, which might have played a role in our high enrollment rate.<br>• Reasons for *not enrolling*: too demanding study to incorporate into a daily routine (*n* = 5) and fear of blood extraction (*n* = 1). These 6 potential participants resided all in Guatemala City with likely a more stressful life (longer trips to school/training facilities), 3 were elite athletes (tier 4, McKay et al., 2022).<br>• Delay *from enrollment to starting the study*: until the onset of the next menstrual cycle (~1 – 4 weeks). If enrolled during menstruation or became ill during the first days of the cycle, the study was postponed, but participants began documenting at least basal body temperature (BBT).<br>• Reasons for *not starting the study*: limited access to communication, i.e., internet and mobile phone signal (*n* = 1) and emotional instability (*n* = 1)
**Keeping participants in the study**<br>*Reasons for…*	• *Dropouts*: overwhelmed with school commitments (*n* = 1); did not return diet log + oligomenorrhea (studied cycle = 62 days) + lost interest in BBT log (*n* = 1)<br>• *Repeating the study in a second cycle of the same participant*: Athletes learned why we were doing the study and received individual feedback about their diet and ovulatory status. Thus, some chose to repeat the study in a subsequent cycle if self-reported EI was not representative of the follicular phase of the studied cycle (*n* = 1), had an ovulatory disturbed cycle (*n* = 2), had a BBT chart difficult to interpret (*n* = 1) and if we failed to quantify their progesterone within the peak period (*n* = 3).
**Exclusions**	• We *failed to quantify progesterone* within the peak period (*n* = 5) despite 1 – 2 attempts per athlete, including two false LH positives. The cycles with false LH-positive results were longer than expected (32 – 42 days). According to reference ranges of the laboratories and BBT quantitative interpretation, the progesterone concentration (0.32 – 0.40 $ng \cdot mL^{-1}$, quantified 7 – 8 days after LH detection) in these cycles was not evidence of anovulation but indicative of testing before the ovulatory period. We highlight that 80 – 100% of the 2 – 5 menstrual cycles observed in each of these participants were ovulatory disturbed as per BBT quantitative interpretation.
Reasons for further exclusions	• Reported a *fever* the night before blood sample collection (*n* = 1)<br>• Self-reported *EI* as *not representative of the studied follicular phase* (*n* = 1)<br>• *Missed 1 day of diet register* (*n* = 1), and<br>• *Described dietary intake superficially* throughout the weekend (*n* = 1)
**Adherence**	• *Forgot occasionally to measure BBT* (33%) *or to self-perform 'ovulation' test* in urine (13%)<br>• *Failed to refrain from late night plus alcohol consumption during the critical period of BBT interpretation* (*n* = 1); nevertheless, we confirmed in the following menstrual cycle how a late night with alcohol ingestion was affecting her BBT and managed to interpret with 2 quantitative methods in agreement to progesterone concentration.<br>• *Most demanding task:* weighed and photographed food diary. Participants were happy after finalizing the diet log or tired in the last few days, especially if it coincided with the weekend.

TABLE S3 | Suggestions for future research.

**Screening**	• Besides the LEAF-Q (Melin et al., 2014), use a validated tool to identify 'restricted eaters'.<br>• For exclusion purposes and better scheduling of hormonal assessments, record prospective counts of menstrual cycle length with basal body temperature (BBT) monitoring.<br>• Discriminate for insulin resistance (fasting glucose + insulin) and polycystic ovary syndrome.<br>• If beginning with eumenorrheic participants, confirm the absence of ovulatory disturbances with an adequate increase of progesterone in the luteal phase.
**Ideal scenario**	• Hormonal disruptors should be ideally eliminated from diet and environment.
**Basal body temperature**	• Instruct participants about 1) the technique to measure BBT and 2) how to detect mistakes or low battery in the device and repeat the measurement immediately. Instruct them to make sure they turn off the thermometer after recording data and offer a second device as a backup.<br>• Have participants send a photo of the screen of the digital thermometer by phone app and build up the BBT chart daily. Considering the expected cycle length and how the previous chart(s) look might help to identify that the luteinizing hormone (LH) peak is about to occur.<br>• To reduce the burden for the athlete with BBT log with notes on factors that may affect BBT, teach them what is it that they need to report along with the image of the measurement, and ask questions on the spot as needed.
**Energy availability** (EA) in ***observational studies***	• Assess diet quality with a validated tool. Estimate EA from day 3 to day 9 of the menstrual cycle. Have sufficient kitchen scales; also, dietitians and trained staff observe, code, verify food portions, and analyze diet right away. Ask for photographs of all food and drinks consumed during the cycle.<br>• Record training during the full menstrual cycle but use the same days of the weighed dietary log in the EA estimation. Monitor body mass weekly in equal conditions with a precision of ≤50 grams.<br>The above shall help decide objectively if the estimate of EA is indeed representative of the follicular phase and the luteal phase of the studied menstrual cycle.<br>• Validate energy intake. Monitor HRV.<br>• Analyse diet composition in combination with the theoretical metabolic impact of exercise on the ovulatory status. Estimate carbohydrate availability as defined by Loucks (2004): carbohydrate intake minus carbohydrate oxidized during EEE normalized to FFM.
EA in ***interventional studies***	• Use BBT and urinary kits for LH and metabolites of oestrogen and progesterone to confirm the absence of ovulatory disturbances. These kits may be sent by post to 'naturally menstruating' potential participants after questionnaire screening. Follow potential participants via phone apps.<br>• Study several amounts of EA, e.g., 45, 40, 35, 30, and 25 kcal·kg $FFM^{-1}\cdot day^{-1}$, prescribing the same amount during at least 2 menstrual cycles, and follow up the cycle after the intervention with the assessments of EA during the follicular phase and hormonal adequacy during the luteal phase. Sufficient staff will be needed to keep each case in check. Ideally, all food should be served or at least provided. Monitor body mass weekly in equal conditions with a precision of ≤50 grams.<br>• The prescribed diet should meet protein and micronutrient recommendations.<br>• Different diet compositions could be tested, i.e. low carbohydrate with and without dietary restriction. Monitor HRV.<br>• Besides controlling EEE, assess its metabolic impact to be able to estimate carbohydrate availability defined as by Loucks (2004): carbohydrate intake minus carbohydrate oxidized during EEE normalized to FFM.
**LH detection or quantification**	• Ideally, quantify urinary LH and metabolites of oestrogen and progesterone. If not, use qualitative urinary kits. Qualitative urinary detection of only LH may be enough when following further advice.<br>• Use the expected shortest cycle as recorded by the participant to decide upon the day of the cycle to start testing, not the average length of previous cycles.<br>• If the BBT chart shows no sign of ovulation after the expected LH peak period, keep on testing daily as needed! Have more than enough LH tests!

(continued)

TABLE S3 | (continued)

**Serum progesterone**	• Test on days 6 – 7 after the day with the last LH positive and not until day 9!<br>• If expecting a short luteal phase, plan to test on day 3 and day 6 after the last consecutive day with positive LH detection; do not lose all data if 1 – 2 assessments are planned! The shortest luteal phase we documented was 4 days –LH positive on day 22 during a 27-day cycle (excluded from our analysis due to lack of progesterone evidence).
**Follow up**	• At least interpret the BBT of 1 menstrual cycle after the last cycle studied.
**Data analysis**	• Separate for the type of ovulatory disturbance (anovulation, short luteal phase, or luteal deficiency); indicate the ovulatory status of oligomenorrheic cycles as they may or not be ovulatory disturbed.

LEAF-Q: Low energy availability in females questionnaire; HRV: heart rate variability; EEE: exercise energy expenditure; FFM: fat-free mass
